# Knowledge and attitudes towards complementary and alternative medicine among medical students in Turkey

**DOI:** 10.1186/1472-6882-12-115

**Published:** 2012-08-03

**Authors:** Hulya Akan, Guldal Izbirak, Elif Çiğdem Kaspar, Çiğdem Apaydin Kaya, Serpil Aydin, Nejat Demircan, P Gamze Bucaktepe, Cahit Özer, Hüseyin A Sahin, Osman Hayran

**Affiliations:** 1 Department of Family Medicine, Yeditepe University Faculty of Medicine, İnönü Mahallesi, Kayışdağı Cad., 26 Ağustos Yerleşimi, Kadıköy, İstanbul, 34755, Turkey; 2 Department of Biostatistics, Yeditepe University Faculty of Medicine, İnönü Mahallesi, Kayışdağı Cad., 26 Ağustos Yerleşimi, Kadıköy, İstanbul, 34755, Turkey; 3 Department of Family Medicine, Marmara University Medical Faculty, Mimar Sinan Cad. Kaynarca, Pendik, İstanbul, Turkey; 4 Department of Family Medicine, Adnan Menderes University Faculty of Medicine, Aydin, Turkey; 5 Department of Family Medicine, Zonguldak Karaelmas University Faculty of Medicine, Esenkoy-Kozlu, Zonguldak, 67600, Turkey; 6 Department of Family Medicine, Dicle University Faculty of Medicine, Diyarbakir, Turkey; 7 Department of Family Medicine, Faculty of Medicine, Mustafa Kemal University, Hatay, 31100, Turkey; 8 Department of Family Medicine, Van Yüzüncü Yıl University Faculty of Medicine, Merkez Van, Turkey; 9 Department of Public Health, Yeditepe University Faculty of Medicine, İnönü Mahallesi, Kayışdağı Cad., 26 Ağustos Yerleşimi, Kadıköy, İstanbul, 34755, Turkey

## Abstract

**Objective:**

This study aims to examine knowledge and attitudes towards Complementary and Alternative Medicine among medical students in Turkey, and find out whether they want to be trained in Complementary and Alternative Medicine (CAM).

**Methods:**

A cross-sectional study was carried out between October and December 2010 among medical students. Data were collected from a total of seven medical schools.

**Findings:**

The study included 943 medical students. The most well known methods among the students were herbal treatment (81.2 %), acupuncture (80.8 %), hypnosis (78.8 %), body-based practices including massage (77 %) and meditation (65.2 %), respectively. Acupuncture, aromatherapy, herbal treatment and meditation were better known among female participants compared to males (p < 0.05). Females and first year students, generally had more positive attitudes. A larger proportion of female students compared to male students reported that a doctor should be knowledgeable about CAM (p = 0.001), and this knowledge would be helpful in their future professional lives (p = 0.015). Positive attitudes towards and willingness to receive training declined as the number of years spent in the faculty of medicine increased.

**Conclusions:**

Majority of the medical students were familiar with the CAM methods widely used in Turkey, while most of them had positive attitudes towards CAM as well as willingness to receive training on the subject, and they were likely to recommend CAM methods to their patients in their future professional lives. With its gradual scientific development and increasing popularity, there appears a need for a coordinated policy in integrating CAM into the medical curriculum, by taking expectations of and feedback from medical students into consideration in setting educational standards.

## Background

The National Center for Complementary and Alternative Medicine (NCCAM) defines Complementary and Alternative Medicine (CAM) as “medical and health systems, applications and products currently not considered as part of conventional medicine” [1].

An increased interest in CAM is observed among both the general population and health professionals. Despite its rising popularity, CAM has been excluded from conventional medical training for many years, however recently there is a tendency to include it in the medical curricula in some countries [2]. CAM began to draw attention among medical circles in 1990 when it was found that 13.7 billion US dollars were spent on CAM applications and that one in three Americans made use of CAM in 1993 [3]. A follow-up study by the same research group showed that use of alternative medicine increased by 65 % in 1997, with an increase in spending by 45.2 % [4]. It appears that CAM gained increasing popularity among medical trainers and students [5-7].

Similarly, use of CAM methods has grown in popularity in Turkey. Surveys with patient subgroups have shown that many patients with chronic diseases have used at least one of the CAM methods. Most commonly used methods were herbal treatment, massage and acupuncture, but they shared this information rarely with their primary doctors. Most of these studies indicated that it is important for healthcare professionals to discuss and give counseling about use of CAM to their patients [8-16].

In 1996, a broad-based panel including medical and nursing schools and representatives from the American Medical Association (AMA), American Academy of Family Practice (AAFP), Association of American Medical Colleges (AAMC), Federation of State Medical Boards, Pew Health Professions Commission, American Medical Student Association (AMSA), and other organizations gathered to make an evaluation on CAM education, and proposed CAM’s integration into the curricula by the discipline with its philosophical underpinnings, scientific bases, educational preparation, applications, and evidence concerning its reliability and effectiveness [17]. In some countries, medical curricula already have structured programs on complementary and alternative medicine. Studies about medical students’ attitudes toward and knowledge of CAM in several countries usually showed positive attitudes towards and a high level of desire to learn about CAM in medical schools.

In Turkey, practices of CAM vary widely regarding inclusion of CAM in the medical curriculum in the absence of a nationally agreed policy. Also, there are very few studies on students’ attitudes toward, knowledge and desire to learn about CAM. Two studies with medical and nursing students were local studies included only one medical school [18,19]. Another study carried out with general practitioners concluded that general practitioners wanted to learn more about CAM, and improve their knowledge [20]. So, there is lack of data at the national level. Therefore, this study aimed to examine knowledge and attitudes towards Complementary and Alternative Medicine among students attending faculties of medicine in Turkey, and find out whether they want to be trained in Complementary and Alternative Medicine.

## Methods

A cross-sectional study was carried out between October and December 2010 among medical students. In Turkey, there are 64 medical faculties, of which 54 are public, and a total of 34.869 students were attending these faculties at the end of 2009. Study population included 1st year, 5th year and 6th year medical students. For data collection, all provinces of Turkey were divided into three sub-groups on the basis of economic development level as measured by the State Planning Organization in 2009, and two faculties of medicine were randomly selected from each sub-group. Data were collected from a total of seven faculties of medicine, including six randomly selected faculties and Faculty of Medicine of Yeditepe University where the pilot study was carried out. The number of students required to represent the population of the study was 348 with a 95 % confidence level. For cluster sampling, we targeted to double the number of participants, and data were collected from 1st year, 5th year, and 6th year students attending the Faculties of Medicine in order to be able compare first-year undergraduates with intern students.

Students completed the questionnaires during class hours after obtaining necessary permits from the Faculty administrations. The questionnaire used was prepared by the researcher after review of the relevant international literature, and finalized following a pilot application prior to data collection.

The questionnaire consisted of three parts. The first part consisted of questions on sociodemographic characteristics of the students, such as age and gender. The second part consisted of yes-no questions asking the students whether they were familiar with the sixteen CAM methods that were selected based on a consensus of the study group after screening Turkish websites on CAM ; what they thought about the effectiveness of these methods; whether they would like to be trained in these methods; and whether they will be recommending these methods to their patients in their future professional lives. The third part consisted of 7-point Likert-type items, aiming to identify attitudes towards CAM designed on the basis of the questions used by Furnham in his 2003 study[7], after obtaining appropriate permission from the author. The research was approved by the Ethics Committee of Yeditepe University.

### Data analysis

Statistical analyses were performed using SPSS v.19. Descriptive analyses and relevant significance tests were used for comparisons. The level of significance was set at p <0.05.

## Results

Data were collected from a total of 943 medical students from seven faculties of medicine. Of these participants, 413 were female, and 511 were male, and 19 did not identify their gender. The mean age was 20.6 ±2.7 years. The most well known methods among the students were herbal treatment (n = 765, 81.2 %), acupuncture (n = 762, 80.8 %), hypnosis (n = 743, 78.8 %), and manipulative and body-based practices including massage (n = 726, 77 %) and meditation (n = 615, 65.2 %), respectively. Less than 10 % of the participants were familiar with homeopathy, chiropractic, the Alexander Technique, reflexology, shiatsu and chigong. Acupuncture, aromatherapy, herbal treatment and meditation were better known among female participants compared to males (p < 0.05), and a larger proportion of male participants were more familiar with chiropractic than females (p < 0.005). A larger proportion of the 5th and 6th year students reported familiarity with bioenergy (p = 0.009), chelation (p = 0.000), neural therapy (p = 0.009), ayurveda (p = 0.023) and homeopathy (p = 0.042) compared to 1st year students. There were no significant differences between the grades in the most well known five methods in the study sample. Table 1 shows distribution of opinions on the effectiveness of the five most well known methods among students, their willingness to be trained in these methods, and future likelihood of recommending them to their patients.

**Table 1:** Percentage distribution of answers to other questions given by participants who knew about Alternative Medicine Methods (n = 943)

	**I know %**	**Believe effectiveness %**	**I want education %**	**I will suggest to my patients %**
**Herbal Treatment**	81,2	79,4	66,6	70,6
**Acupunture**	80,8	74,5	61,2	63,4
**Hypnosis**	78,8	63,8	65,8	48,2
**Body work (including massage)**	77	77,8	60,9	66,4
**Meditation**	65,2	63,6	53,8	52,2

The only inter-gender difference in effectiveness was related with the method of meditation, with a larger proportion of the female participants reporting it as an efficient method. A larger proportion of the 1st year students believed that acupuncture, herbal treatment and manipulative and body-based practices including massage were effective methods compared to 5th and 6th year students (p < 0.005). Opinions on the effectiveness of the remaining methods did not vary by grade.

A larger proportion of female participants reported that they would recommend acupuncture (p = 0.003) and ayurveda (p = 0.019) compared to males, and future likelihood of recommending other methods did not vary by gender. A larger proportion of the 1st year students reported that they would recommend herbal treatment, acupuncture, manipulative and body-based practices including massage, meditation, shiatsu and ayurveda compared to 5th and 6th year students (p < 0.005); and future likelihood of recommending other methods did not vary by gender. A larger proportion of the female participants expressed their willingness to be trained in acupuncture (p = 0.008) and meditation (p = 0.004) compared to males. Willingness to receive training in other methods did not vary by gender. In all methods except for homeopathy, a larger proportion of the 1st year students reported that they would like to receive training compared to 5th and 6th year students (p < 0.05).

Overall, attitude towards CAM among the students was positive; they believed that knowledge of CAM would be useful, and current CAM practitioners were not well-trained, and they had to be medically qualified. Overall, students believed that CAM should be taught in medical schools, and doctors should be familiar with CAM treatments, and knowledge of CAM would be useful in their future professional lives. Table 2 shows attitudes with the highest and lowest percentages of agreement.

**Table 2:** Attitude questions with the highest (mean score > 5.00) and lowest (mean score < 3.00) mean scores (n = 943)

**Items**	**Mean ± S.D**
All practitioners of CAM should be medically qualified.	5,37 ± 1,85
I am intereseted in exploring new teratment modalities.	5,28 ± 1,80
Women have more tendency to CAM than men.	5,20 ± 1,9
On avarage, practitioners of CAM make less money than other doctors.	2,93 ± 1,86
I beilieve in alternative approaches in health area	2,92 ± 1,85
Most Practitioners of CAM receive a thorough training.	2,88 ± 1,65
Much of CAM is actually dangerous to the health of the patients.	2,88 ± 1,66
Treating a condition using CAM is safer than using modern methods	2,69 ± 1,71
You need to be “gifted” to carry out CAM	2,25 ± 1,61
One person of my family is currently using CAM treatment	2,17 ± 1,9

Students who had a personal interest in CAM; who thought it as an important aspect of medical practice; those who had a family member currently receiving CAM treatment; who thought that modern medicine had limitations of its own; who thought that patients had a right to choose between modern medicine and CAM; and, who thought that spiritual phenomena have an effect on health were of the opinion that CAM modalities should be included in the medical curriculum (p < 0.005).

Significant inter-gender differences were found in attitudes in relation to five questions only (Table 3). A larger proportion of the female students compared to male students reported that a doctor should be knowledgable about CAM (p = 0.001), and it would be helpful in their future professional lives (p = 0.015). A larger proportion of male students reported that CAM is more of an art than science (p = 0.026), patients have the right to choose between modern medicine and CAM (p = 0.004), and they believed in alternative approaches to health (p = 0.000). Table 4 shows inter-grade differences in the Likert-type attitude questions: 1st year students had higher mean scores for most of the items, whereas 5th year and 6th year students expressed more agreement with the following statements: CAM is underestimated in the world of medicine; it is effective in the treatment of minor complaints and illnesses only; it is certainly non-scientific and vague; and, use of CAM treatments are actually detrimental for the health of patients (p < 0.005).

**Table 3:** Attitudes in which there were significant inter-gender differences

	**Men (n = 511)**	**Women (n = 413)**	**p**
CAM is more art than science (q18)	3,78 ± 1,82	3,51 ± 1,78	0,026*
A doctor should know CAM methods (q27)	4,46 ± 1,97	4,92 ± 1,75	0,001*
Knowledge of CAM is needed in my future professionalism. (q28)	4,20 ± 2,03	4,61 ± 2,37	0,015*
I believe in that patients have right to choose between modern medicine and CAM (q36)	3,79 ± 2,12	3,38 ± 2,03	0,004*
I believe in alternative approaches in medicine (q37)	3,16 ± 1,96	2,63 ± 1,67	0,000*

Mann–Whitney U test p values, *p < 0,05.

**Table 4:** Distribution of the Items of the Scale on Attitudes towards Alternative Methods in Medicine by Grade (n = 943)

**ITEMS**	**First year**	**5. and 6. class**	**p**
	Mean ± SD	Mean ± SD	
All practitioners of CAM should be medically qualified.	5,51 ± 1,75	5,15 ± 1,98	0,024*
On avarage, practitioners of CAM make less money than other doctors.	3,12 ± 1,88	2,70 ± 1,89	0,000*
CAM has low status within medicine	4,14 ± 1,78	4,57 ± 1,85	0,001*
CAM is only effective in treating minor complaints.	3,91 ± 1,77	4,48 ± 1,94	0,000*
CAM is fairly unscientific.	3,09 ± 1,84	3,47 ± 2,05	0,014*
CAM has advanced considerably in recent years in understanding of illness and diseases	4,16 ± 1,65	3,52 ± 1,8	0,000*
Practitioners of CAM are more prepared to listen to their patients.	4,56 ± 1,73	4,21 ± 1,86	0,012*
Patients on CAM hardly ever get better	3,15 ± 1,57	3,84 ± 1,72	0,000*
Despite considerable research, there are few aplicable results in CAM.	3,85 ± 1,58	4,66 ± 1,80	0,000*
CAM should be thught in medical school.	4,66 ± 2,00	4,12 ± 2,13	0,000*
Women have more tendency to CAM than men.	5,93 ± 1,91	5,58 ± 1,70	0,000*
Most practitioners of CAM receive a thorough training.	3,03 ± 1,65	2,59 ± 1,63	0,000*
CAM is safer than modern medical treatments	2,76 ± 1,70	2,55 ± 1,72	0,027*
You need to be “gifted” to carry out CAM	2,38 ± 1,65	2,00 ± 1,51	0,000*
A surprising number of patients claim its effective at curing their illness.	4,44 ± 165	4,14 ± 1,88	0,027*
CAM is more cost-effective than modern medicine	3,47 ± 1,70	3,21 ± 1,79	0,020*
Much of CAM is actually dangerous to the health of patients	2,75 ± 1,58	3,14 ± 1,78	0,003*
The reason for the success of CAM is mainly due to treating the whole person.	3,73 ± 1,73	3,19 ± 1,72	0,000*
A doctor should know CAM methods	4,95 ± 1,73	4,13 ± 2,08	0,000*
Knowledge of CAM is needed in my future professionalism	4,67 ± 2,21	3,83 ± 2,08	0,000*
I believe that to suggest CAM modalities to their patients is responsibility of doctors.	4,20 ± 1,91	3,22 ± 2,04	0,000*
I am personally interested in CAM	4,16 ± 2,03	3,53 ± 2,14	0,000*
CAM is an important part of my professionalism	3,59 ± 1,95	2,80 ± 1,92	0,000*
CAM is an important part of my culture	3,52 ± 2,01	3,17 ± 1,95	0,013*
I believe that CAM may have positive effect on general health outcomes	4,25 ± 1,82	3,71 ± 1,84	0,000*

Mann–Whitney U test p values, *p < 0,05.

## Discussion

A majority of the medical students in the study population were familiar with some of the CAM methods while some were almost unheard of. Studies on the general population show that among the methods included in this study, herbal treatment, manipulative and body-based practices including massage and acupuncture are the most well known and the most frequently practiced modalities [8-16,21-24].

Medical students are also a part of the general population, and it is only natural, especially for 1st year students, to know about the most well-known and most frequently practiced methods among the general population. Fifth and 6th year students were more knowledgeable about the methods that are less known to the study sample compared to 1st year students. None of the medical schools included in the study had a structured CAM training. Thus, it might be the case that 5th and 6th year students learn about less known methods from patients during their clinical practice or through sharing of experiences [7].

Overall, students were more likely to think that methods that they were familiar with were effective, and they were more likely to recommend these methods to their patients in their future professional lives. It was surprising that students expressed their wish to be trained on these methods independent of their familiarity.

There were inter-gender differences in terms of the CAM methods known, opinions on the effectiveness of different methods, methods in which they wanted to be trained, and attitudes towards the methods. Female participants were more familiar with the most well known methods in the whole study group and had more positive attitudes toward CAM.

Previous studies in different countries have also found that female have more positive attitudes towards CAM, and are more likely to use it [25-28]. As in the whole world, increasingly more female students have been choosing to study medicine than male students in Turkey. We can assume that this gender difference may increase medical students’ willingness for training on CAM in the future.

Many studies show that medical students have positive attitudes towards CAM, and they support its inclusion in the medical curricula [5-7,29,30]. Despite increasing interest in CAM, many faculties of medicine do not provide structured CAM training, and those that do provide it have very different teaching goals and content [31-34].

Only a few studies were carried out in Turkey on the knowledge and attitude of medical students towards CAM. In 2004, Uzun&Tan found that 64 % of nursing students of all grades reported that CAM could be included in the curriculum, and 62.3 % reported that it could be used in practice.[19] Nursing students had generally a more positive attitude than medical students. In a study conducted in 2006–2007 with the participation of medical students and nursing students of all grades at Ege University, Yıldırım *et al*. used questions developed on the basis of literature, and found that 61.3 % of the nursing students and 37.9 % of the medical student thought that CAM should be included in the school curriculum [18]. The difference might have been related to the methodologic differences in nursing and medical schools. Unlike our study representing all regions of Turkey, their study was carried out in a university from the Western part of Turkey, which was reported as one of the limitations of their study by the authors. Another study with general practitioners in Bursa, a city in the western part of Turkey, found that 62.7 % of the practitioners believed that a CAM training should be provided in faculties of medicine [20]. The present study can be considered complementary to these findings, showing that medical students in Turkey believe that CAM should be included in the curriculum, and that knowledge of CAM would be useful in their future professional lives, and doctors should be familiar with CAM methods. Many factors can be involved in shaping attitudes of the students, including environmental and individual factors such as having a family member who receives CAM treatment, having a personal interest in CAM, believing that spiritual phenomena have an effect on health, and belief in a patient’s right to choose between modern and alternative medicine. Overall, positive attitudes towards and willingness to receive training in CAM methods declined as the number of years spent in the faculty of medicine increased [7,35]. This change of attitude probably results from increased exposure to evidence-based medicine, trainers who act as role models, and personal experiences with the patients.

CAM applications make up a significant portion of health spending, and there is an increasing demand for CAM applications. Medical doctors, even if they are not CAM practitioners themselves and have negative attitudes towards CAM, will from time to time feel the need to be able to provide guidance for their patients and be familiar with CAM methods at least at a minimal level for drug interactions and treatment effects [32]. It seems to be the case that in future medical students will show a more strongly demand to receive training in this field. On the other hand, CAM is a very broad field with many different applications and despite increasing number of scientific research on some methods, not all methods are evidence-based. It is difficult to include all CAM applications into the curriculum, and locate them into the medical programme, and determine the level of training to be provided.

Our study was limited in some respects. Because it had a cross-sectional design, it was not possible to analyze the factors that have an effect on attitudes over time. A different, prospective, and interventional design is required to interpret how current medical education and sociocultural factors have an impact on students’ knowledge and attitudes. Although overall response rate was over 93 % and only a few students declined to complete the questionnaire, students with a negative attitude and less familiarity with CAM might have not answered the questionnaire and this may have affected some results. Because of high response rate and achievement to reach to the targeted number of students, we think this bias may have had some effect on overall results.

## Conclusions

Majority of the medical students were familiar with the CAM methods widely used in Turkey, and knowledge about the methods which were less frequently used has been increasing with the senior years in the medical faculty, even though these methods are not included in the curriculum. Overall, most students had positive attitudes towards CAM; they wanted to receive training on the subject; and they are likely to recommend CAM methods to their patients in their future professional lives. Currently, none of the medical curricula in Turkey has a structured standard CAM training. With a gradual scientific development and increasing popularity, there appears a need for a coordinated policy in integrating CAM into the medical cirruculum, and expectations of and feedback from medical students should be taken into consideration in setting educational standards.

## Competing interests

The authors declare that they have no competing interests.

## Authors' contributions

HA carried out the design and coordination of the study and data entry and analysis. HA also participated in the sequence alignment and drafted the manuscript. GĐ carried out the data entry and analysis and in the sequence alignment and drafted the manuscript. EK carried out statistics and also conducted data entry. ÇA, SA, ND,GE, CÖ, HAS carried out the study in their regions. OH designed the methodology of the study and performed the statistical analysis. OH participated in its design and coordination and helped to draft the manuscript. All authors read and approved the final manuscript.

## Pre-publication history

The pre-publication history for this paper can be accessed here:

http://www.biomedcentral.com/1472-6882/12/115/prepub
